# Direct observation of nuclear reorganization driven by ultrafast spin transitions

**DOI:** 10.1038/s41467-020-15187-y

**Published:** 2020-03-23

**Authors:** Yifeng Jiang, Lai Chung Liu, Antoine Sarracini, Kamil M. Krawczyk, Jordan S. Wentzell, Cheng Lu, Ryan L. Field, Samir F. Matar, Wojciech Gawelda, Henrike M. Müller-Werkmeister, R. J. Dwayne Miller

**Affiliations:** 1 Max Planck Institute for the Structure and Dynamics of Matter, Luruper Chaussee 149, 22761 Hamburg, Germany; 2 Departments of Chemistry and Physics, University of Toronto, 80 St. George St., Toronto, M5S 3H6 ON Canada; 3 Lebanese German University, LGU, Sahel-Alma, P.O. Box 206, Jounieh, Lebanon; 4 European XFEL, Holzkoppel 4, 22869 Schenefeld, Germany; 5 Faculty of Physics, Adam Mickiewicz University, ul. Uniwersytetu Poznańskiego 2, 61-614 Poznań, Poland; 6 Institute of Chemistry, University of Potsdam, Karl-Liebknecht-Str. 24-25, 14476 Potsdam-Golm, Germany; 7 Present Address: European XFEL, Holzkoppel 4, 22869 Schenefeld, Germany; 8 Present Address: Uncharted Software, 600-2 Berkeley St., Toronto, M5A 4J5 ON Canada

**Keywords:** Ligands, Solid-state chemistry, Photocatalysis, Excited states

## Abstract

One of the most basic molecular photophysical processes is that of spin transitions and intersystem crossing between excited states surfaces. The change in spin states affects the spatial distribution of electron density through the spin orbit coupling interaction. The subsequent nuclear reorganization reports on the full extent of the spin induced change in electron distribution, which can be treated similarly to intramolecular charge transfer with effective reaction coordinates depicting the spin transition. Here, single-crystal [Fe^II^(bpy)_3_](PF_6_)_2_, a prototypical system for spin crossover (SCO) dynamics, is studied using ultrafast electron diffraction in the single-photon excitation regime. The photoinduced SCO dynamics are resolved, revealing two distinct processes with a (450 ± 20)-fs fast component and a (2.4 ± 0.4)-ps slow component. Using principal component analysis, we uncover the key structural modes, ultrafast Fe–N bond elongations coupled with ligand motions, that define the effective reaction coordinate to fully capture the relevant molecular reorganization.

## Abstract (web summary)

Electron spin is a fundamental property of molecules, and changes in spin state affect both molecular structure and dynamics. Here, the authors resolve, by ultrafast electron diffraction, the nuclear reorganization stabilizing spin transitions in a [Fe^II^(bpy)_3_](PF_6_)_2_ crystal.

## Introduction

**Fig. 1:**
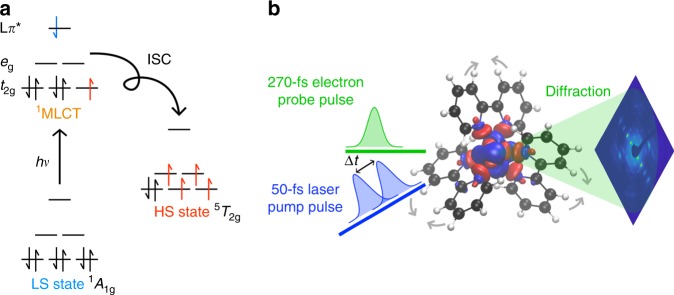
SCO and schematic of the ultrafast electron diffraction setup. **a** Schematic electronic configurations for the SCO model system BPY following the Pauli principle and Hund’s rules^11^. After the initial photoexcitation from the low spin (LS) state into the MLCT state, the system decays to the high spin (HS) state through a number of intersystem crossings (ISC). **b** Schematic of the experimental beam interactions using variable time delays (Δ*t*) to catch the structural changes. The driving force is the change in electron density during SCO as shown in the figure. Single crystal BPY was investigated by ultrafast electron diffraction in a pump-probe experiment with 400-nm excitation and 95 keV electron probe pulses. The time resolution achieved is (270 ± 30) fs FWHM. Gray arrows show the changes in the nuclear configuration. Red denotes positive values for the electron density differences during SCO, and blue denotes the negative value for the electron density differences (see Supplementary Fig. 1).

Electron spin is one of the most fundamental quantities defining the properties of molecules. In this regard, changes in spin state have been extensively studied. A detailed understanding of these processes is particularly important in the development of advanced materials for applications from high-speed memory storage to light-harvesting materials^1–5^. Questions remain on how spin state changes directly affect molecular structures and dynamics. A proper theoretical description of this process can only be achieved with a full relativistic treatment of spin orbit coupling using the Dirac equation^6,7^. Within this theoretical framework, it is possible to describe the experimentally derived Pauli exclusion principle and Hund’s rule^8,9^. Microscopically, it is the fluctuation of magnetic fields associated with these relativistically correlated electron motions around the nuclei that introduces the quantized unit of an oriented spin state^8,10^. It is this intrinsic correlation through the magnetic fields associated with electron spin that accounts for the relationship between spin states and the spatial distribution of electron density as stated in Hund’s rule. This fundamental relationship can be directly probed in the study of structural changes of spin crossover (SCO) materials. SCO refers to a reversible phase transition between a low-spin (LS) state and a high-spin (HS) state that can be triggered by changes in pressure, temperature, and magnetic fields, or by photoexcitation^1–4^. This change in spin states leads to the associated change in electronic distribution across the molecule (Fig. 1), which is coupled in turn to a change in the nuclear configuration seeking the lowest potential energy structure related to the spin-induced change in electron density (see the Supplementary Note 1 and Supplementary Fig. 1)^11,12^. This nuclear rearrangement to stabilize the change in electron distribution can be mapped onto effective reaction coordinates^7,11,13^. These nuclear motions in turn directly report on the changes in electron distribution with changes in spin state. It is, therefore, possible to gain a detailed understanding on how changes in spin states affects the nuclear configuration and the associated macroscopic properties of a material by measuring ultrafast changes in its molecular structure^14,15^.

In this regard, several SCO-active transition metal complexes have been studied as prototypes for light harvesting since they can convert light to chemical energy via photoinduced charge separation^2,16–20^. A major challenge is to find a SCO-active system centered on Earth-abundant iron (as opposed to elements in the platinum group) that has sufficiently long excited-state lifetimes for promoting interfacial charge transfer for energy storage^16,19,20^. This can be achieved to some extent by chemical tailoring of the ligands and solvent to fine-tune the relative energy of metal-centered (MC) states relative to excited charge transfer (CT) states via ligand-field effects. Experimental and theoretical investigations of the spin transitions involved in ultrafast intersystem crossing (ISC) between these electronic surfaces have shown that the rate at which ISC occurs is not dictated solely by the strength of spin-orbit coupling at the metal center^17,18^. To understand the excited state dynamics, the precise nature of the initially excited CT, ligand-centered (LC) or MC states needs to be taken into account^17,18^. These details manifest themselves in the relative energetics of the excited state potential energy surfaces for a given spin state. This information needs to be considered along with the spin-orbit coupling and Franck-Condon (FC) weighted nuclear probabilities that determine the transition probability between electronic surfaces to fully understand the dynamics and degree of structural rearrangements involved in photoinduced SCO processes^17,18^. The design of ligands to gain control over these spin dynamics and material properties has become a research focus^1,15,16,20,21^, which further illustrates the importance of understanding nuclear reorganization processes that stabilize spin transitions.

Towards the aforementioned goal, the mechanisms of photoinduced SCO have been extensively studied to understand the multiple photophysical and structural processes that occur on the femtosecond time scale^2,16^. A prototypical example is the cation iron(II)-tris(bipyridine) ([Fe^II^(bpy)_3_]^2+^) (Fig. 1b)^2,22^. The photophysics of the specific form [Fe^II^(bpy)_3_](PF_6_)_2_ (BPY) have been well characterized, making it ideal for probing the structural rearrangements and correlations associated with the changes in spin state. From X-ray^12,15,22–25^ and optical^26,27^ spectroscopies, it is understood that upon light absorption the molecule is excited from the LS singlet ground state into a metal-to-ligand charge transfer (MLCT) manifold by transferring an electron from the metal center to the ligands. It then relaxes nonradiatively via internal conversion and ISC before arriving into a HS quintet state as a vibrationally hot molecule^17,18,23,25,26^. Rapid intramolecular vibrational energy redistribution (IVR) coupled to vibrational relaxation through energy exchange with the surrounding lattice cools down this state until it eventually reaches a final fully-relaxed HS state^17,18,25,26^. However, the exact pathway with respect to the nuclear motions leading to the nonradiative spin transitions and possible intermediate states remains a subject of ongoing debate^4,26^.

The current interpretation of the structural dynamics of BPY is based on information and extrapolation from other SCO systems that can undergo a thermal LS–HS transition in addition to the photoinduced one. A commonly studied reaction coordinate—the ultrafast elongation of the metal–ligand bond—has been interpreted to be the key structural signature of SCO^23,28–30^. In BPY and other SCO systems^28,30–35^, it has been proposed that this nuclear motion is dynamically coupled to various structural distortions of the ligand. The potential nuclear motions leading to an expanded HS molecule were first discussed in an earlier time-resolved X-ray diffraction (TR-XRD) experiment^36^ and a number of theoretical studies^7,11,13,37^. This nuclear reorganization has since been directly observed by ultrafast electron diffraction (UED) in the related SCO system, [Fe^II^(PM-AzA)_2_](NCS)_2_ (PM-AzA = N-2’-pyridyl-methylene-4-(phenyl-azo)) (AZA)^38^. We note that BPY has been previously studied by TR-XRD^39^ but under extremely high excitation fluence and the results therein are inconsistent with those from other works^26,40–43^. The issue of the laser peak power used in exciting the sample is an important consideration to ensure that the observed photoinduced dynamics can be rationalized with respect to a well-defined initial excited state for SCO without multi-photon effects complicating the state preparation.

The key missing information in understanding the photoinduced processes of SCO is how the atomic nuclei specifically rearrange to stabilize the HS state, which directly probes the nuclear reorganization that stabilizes the spin related changes in electron distribution. This detail further determines the energetics of the SCO process and the relative positions of the spin manifolds in potential energy space along the metal–ligand distance. The dynamics of the process depend on the spin-orbit coupling and Franck-Condon factors that are weighting the spin transition and define the doorway modes between the LS and HS states. Through the subsequent IVR process from the initial FC modes, the system irreversibly undergoes transitions into the lower energy HS manifold (see Supplementary Note 2). It is the change in electron distribution associated with the spin transition that drive the nuclear reorganization. Ideally, one would like to determine these specific motions over the full extent of the molecule to rigorously test theoretical concepts for controlling spin dynamics and tailoring our ability to design molecular systems for real-world applications. In this regard, some previous studies have investigated the role of ligands in photoinduced SCO^44,45^. However, they were not able to directly study the structural dynamics of the spatially extended cooperation between the metal center and the surrounding ligands since this structural information is not readily accessible using optical and X-ray spectroscopic methods. This gap needs to be filled as ligand substitution provides a convenient means to tune SCO processes as well as test our understanding of the factors controlling spin transitions.

In this work, we exploit the simultaneous femtosecond time resolution and high structural sensitivity of UED^14,38^ to directly observe the structural dynamics coupled to the spin transitions in photoinduced SCO in single-crystal BPY, to provide the full extent of the nuclear reorganization process. Electron diffraction resolves the atomic positions of molecules by scattering electrons off the Coulomb potentials of the nuclei to give full structural details^14^. The time-resolved analogue based on pump-probe protocols has opened up the study of atomic motions of photoinduced structural dynamics at the fundamental space-time resolution limits needed to resolve the primary processes of interest^14,46–48^. The important feature of the experiment in the present context is that UED probes the ensemble average of all changes in interatomic distances within the unit cell; the observed evolution of the Bragg peak intensities reflects correlated nuclear motions driven by the photoinduced dynamics^49^. In this study, nuclear motions along the SCO reaction coordinate for BPY are directly observed. These motions can be classified into 4 key reaction modes, which go beyond the expected ultrafast metal–ligand bond elongation. This work establishes a detailed picture of the causal relationship between the electronic spin dynamics of the molecular system and the coordinating ligands. We discuss this coupling between spin and nuclear degrees of freedom at the atomic level by comparing BPY results to those of AZA, rationalizing these differences in terms of the nonradiative relaxation processes and ligand-induced symmetry effects. It is these forces at play that are ultimately related to Hund’s rule in seeking lower energy high spin states.

## Results

### Electron diffraction patterns and structural dynamics

**Fig. 2:**
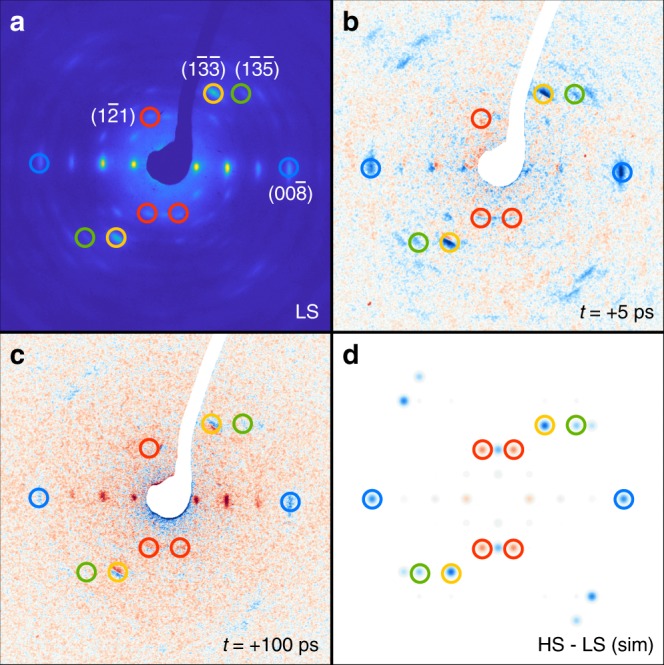
Electron diffraction patterns. **a** Data measured at room temperature (T = 300 K) for the ground state low spin structure. **b** Signal induced by photoexcitation, measured after 5-ps time delay. **c** Signal induced by photoexcitation, measured after 100-ps time delay. **d** Difference between low spin ground state structure determined from X-ray diffraction and high spin structure from our calculation. The colored circles present several selected pairs of Bragg peaks. The positive changes in the intensity of Bragg spots is shown in blue, and the negative changes in the intensity of Bragg spots are shown in red.

The static diffraction pattern of single-crystal BPY at 300 K is shown in Fig. 2a. The experimental difference diffraction pattern observed at +5 ps and at +100 ps after photoexcitation (Fig. 2b, c) are compared to the simulated difference diffraction pattern from the structure factors evaluated from the LS and the HS structures (Fig. 2d). The initial LS ground-state structure is obtained from X-ray diffraction data^50^ whereas the HS one is generated from our structural model^37^ (see Supplementary Note 3).

In most previous UED studies^14,38,47^, the phase transitions of interest could be activated both thermally and optically. Since the thermal and photoinduced states are known to be nearly identical with respect to their spectral features and other properties^14,38,47^, the thermally induced structure is used as the reference for determining the time-dependent evolution of atomic coordinates from the ground to photoinduced structural changes. In the case of SCO, this prior knowledge of the excited state structure can confirm that the observed evolution of the Bragg peak intensities is due to photoinduced SCO. However, this approach is not possible for BPY since its HS state cannot be thermally populated due to its much higher required internal energy relative to the LS ground state. To solve this problem, the reference structure of the photoexcited state is determined by means of a statistical correlation analysis^50^, in combination with ab initio molecular dynamics (AIMD) calculations^37^ to project out the key motions in the evolution from the LS to HS structure (see Supplementary Note 3). We treat the system independent of the lattice, which has been shown to be an excellent approximation. Femtosecond transient absorption spectroscopy studies of the ultrafast dynamics of photoinduced SCO in single-crystal BPY are found to be nearly identical to that in the solution phase^27,41^. In both phases, the photoexcited state remains localized; the extremely fast spin transitions and associated dephasing processes limit the prospect of exciton formation such that the dynamics in the single crystal are similar to that of the isolated molecules in solution.

It is known from static crystallographic studies that BPY has a highly symmetric crystal structure^50^. In the presented analysis, four key structural modes (Fe–N bond elongation (*ξ*_1_), Fe–ligand elongation (*ξ*_2_), Fe–N rotation (*ξ*_3_), and Fe–ligand rotation (*ξ*_4_)) are selected to model the structural dynamics of SCO. Their selection was inferred from previous studies to avoid chemically unreasonable structures^28,37,38^. A model is thus constructed to map the known structural changes between the LS and HS states in solution to those in the single crystal, allowing for a parameterized reconstruction of the evolution of the BPY crystal structure under photoexcitation (see Supplementary Note 3 and Supplementary Table 1). Motion of the PF_6_ counterions are not included as these moieties occupy positions of high symmetry in the unit cell; activation of such modes would generate symmetry-breaking changes to the diffraction pattern (resulting in appearances or disappearances of Bragg peaks) that have not been observed in the UED data. From this model, a HS crystal structure and accompanying structure factors are generated (see Supplementary Table 2). In Fig. 2d, a simulated difference diffraction pattern between the known LS and simulated HS states in the single crystal is shown. In comparison with Fig. 2b, an approximate match can be made between the experimentally measured intensity changes and the simulated ones. Furthermore, the excitation conditions in the present work are within the linear single-photon regime, in a manner consistent with those in previous studies of photoinduced SCO in BPY^26,41^. We conclude that the observed ultrafast changes in Bragg peak intensity herein represent direct structural signatures of photoinduced SCO.

### Short-time evolution of Bragg peak intensity

**Fig. 3:**
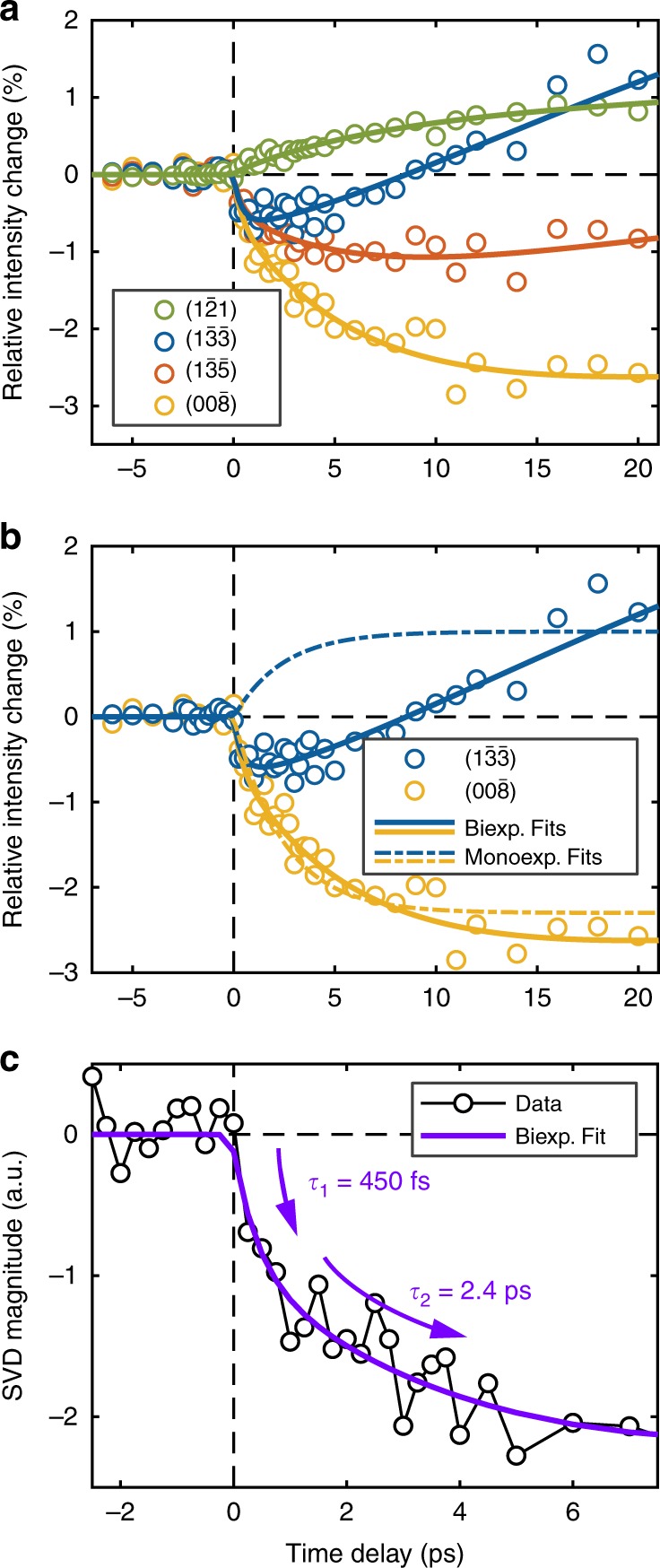
Photoinduced evolution of Bragg spots intensity. **a** Short-time relative change in the intensity of select Bragg spots from *t* = −7 to 20 ps. **b** Global fitting of Bragg spots to show biexponential fit and monoexponential fit of relative intensity changes. **c** These intensity changes of the Bragg peaks are best modeled as a biexponential, with a (450 ± 23)-fs fast component and a (2.4 ± 0.4)-ps slow component. The solid lines represent the global fitting of the evolution of the intensity of the selected Bragg spots.

The photoinduced structural dynamics observed by UED are depicted in Fig. 3. The time traces of prominent Bragg peaks are plotted on the short time scale (from −5 ps to +20 ps) in Fig. 3a. They can be globally and robustly fit to a biexponential function, with a (450 ± 20)-fs fast component and a (2.4 ± 0.4)-ps slow component (Fig. 3b), as opposed to a monoexponential one. The biexponential character is particularly apparent when the time dependence is considered over several diffraction orders. For some Bragg peaks, a monoexponential description is adequate; this simply suggests that the fast and slow components of the underlying biexponential dynamics contribute to different Bragg peaks to varying degrees. Indeed, the time trace of the Bragg peak (1 $\bar 3\,\bar 3$) is particularly representative of the biexponential nature of the observed dynamics; it consists of a rapid decrease followed by a gradual increase in intensity. The plateau observed between +5 ps and +20 ps is indicative of the formation of the final fully-relaxed photoinduced HS state. Further supporting discussions are found in Supplementary Note 4 and Supplementary Note 5 wherein the biexponential fit in this BPY work is contrasted with the monoexponential fit in the previous UED work that studied the AZA molecular system (see Supplementary Fig. 2 and Supplementary Fig. 3).

### Molecular movie presented by four structural dynamics groups

**Fig. 4:**
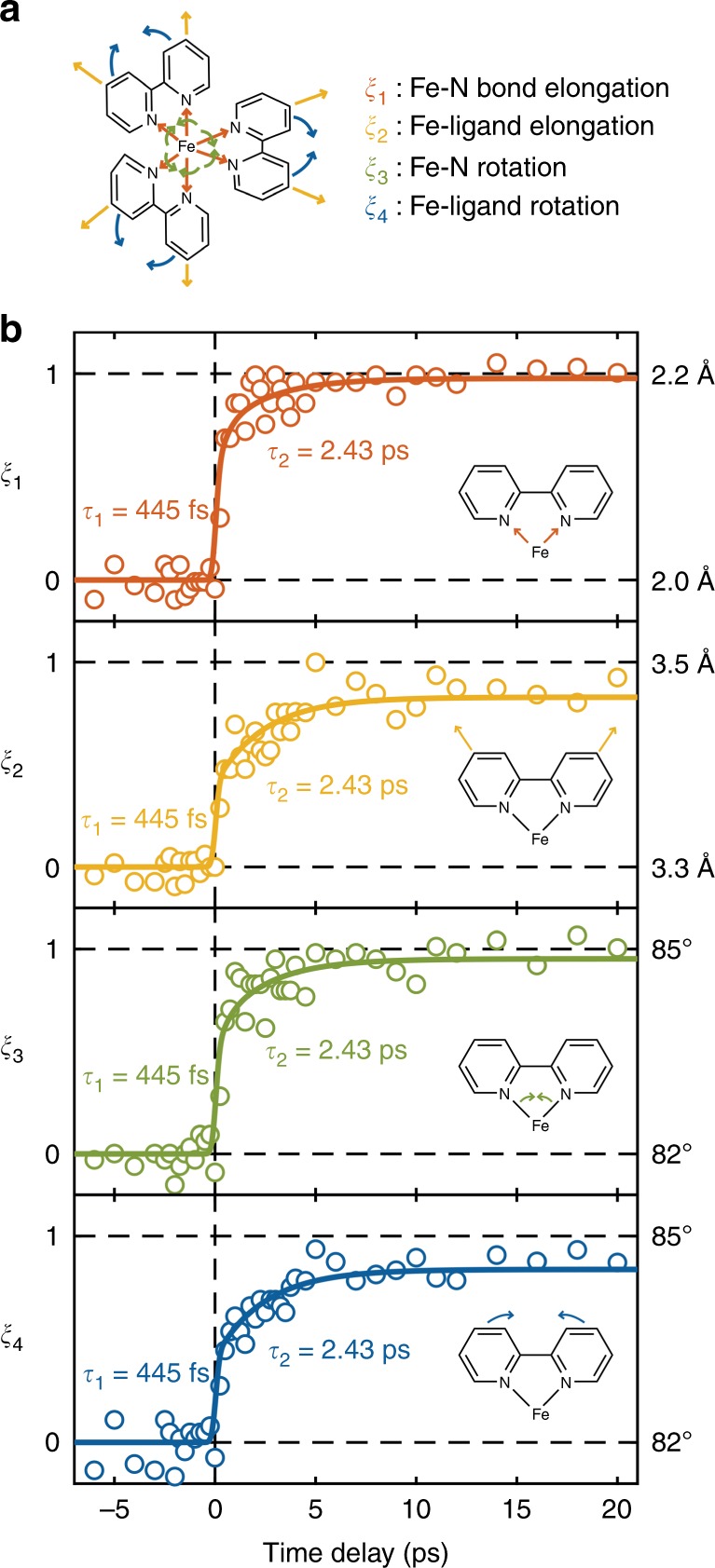
Molecular movie presented by four structural dynamics groups. **a** BPY molecule with four structural dynamics groups. **b** Time-dependence of the refined reaction coordinates of the four structural dynamics group. The solid lines represent the global fitting of the evolution of the four structural dynamics groups. These four key structural dynamics groups are selected to present the full structural dynamics of SCO Fe–N bond elongation *ξ*_1_, Fe–ligand elongation *ξ*_2_, Fe–N rotation *ξ*_3_, and Fe–ligand rotation *ξ*_4_, based on the previous structural studies of SCO. For each time point measured in the UED experiment, a least square fitting routine is used to find the optimal set of parameters of reaction coordinates that would best match the observed changes in diffraction intensity.

Here, a time-dependent map of the molecular motions that occur during the SCO processes in BPY is obtained by the aforementioned parameterized structure model^14,48^. Each of the four parameters—*ξ*_1_, *ξ*_2_, *ξ*_3_, *ξ*_4_—defines a motion and a group of atoms, which are subjected to the forces involved with the spin induced change in electron distribution (see Fig. 4a and Supplementary Note 6). Collectively, they allow for the observed structural dynamics to be decomposed into motions leading from the LS to HS crystal structures. In this analysis, a large variety of candidate structures can be generated in configuration space of the molecule by varying each of the four parameters independently. For each of these candidate structures, a set of structure factors is calculated and compared with those experimentally observed by UED at each time delay. Using the Pearson correlation coefficient as a measure of fit robustness, the molecular structure of the photoexcited state as a function of time is effectively recovered in the form of the optimized time-dependent coordinates *ξ*(t) = (*ξ*_1_(t), *ξ*_2_(t), *ξ*_3_(t), *ξ*_4_(t)), as seen in the four panels of Fig. 4b. In this manner, we have resolved the complete ultrafast structural dynamics of the BPY molecule as it undergoes SCO following photoexcitation. Uniquely, this analysis does not rely on knowing the crystal structure of the HS excited state; instead, it works by sampling a sufficiently large volume of possible configuration spaces in these four reaction coordinates independently to resolve distinct dynamics with potentially different time constants^14,48^.

## Discussion

Globally fitting the four independent structure parameters yielded the same two time constants ((450 ± 20) fs and (2.4 ± 0.4) ps). This suggests that the underlying atomic displacements are coupled, highly correlated motions within the time resolution of our UED measurements. The sub-picosecond component in Fig. 3c is attributed to a combination of Fe–N bond elongation (*ξ*_1_) and ligand torsion (*ξ*_2_ ⊕ *ξ*_3_ ⊕ *ξ*_4_). In another SCO complex, such motions have been previously reported with similar sub-picosecond time constants, 160 fs and 250 fs respectively, and were observed to start immediately upon photoexcitation^28^. Given the (270 ± 30)-fs instrument response time of the present UED setup, such temporal differences would not be resolved. The slower 2.4-ps component is herein assigned to molecular reorganization during the IVR and vibrational cooling leading process to the final fully-relaxed HS structure. This reorganization has been described in the literature^28^ and was observed to be coupled with other motions^19^. However, such restructuring is observed directly as distinct atomic motions in this work. Furthermore, the time constant reported here is in agreement with the vibrational cooling timescales previously described for [Fe^II^(bpy)_3_]^3+^ in solution and the single crystal^26,41^. During the relaxation phase, energy is redistributed to the lattice and other BPY low frequency modes before arrival to the fully relaxed HS excited state structure.

**Fig. 5:**
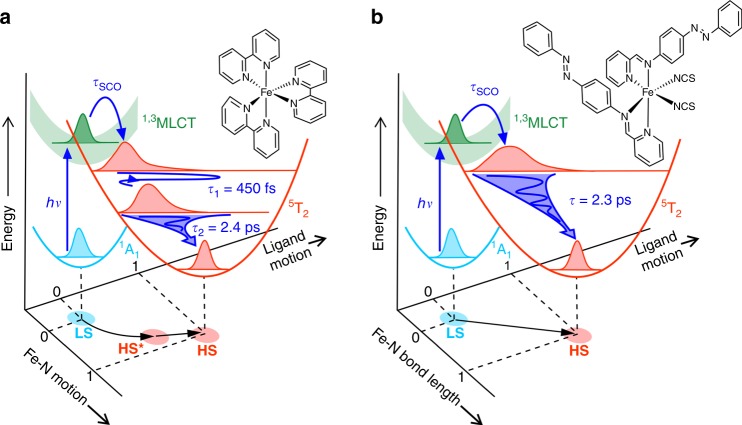
Schematic representation of the proposed reaction pathway. **a** For BPY, photoexcitation is followed by radiationless spin transitions. The vibrationally hot high spin state has a narrow Fe–N bond distribution, which is impulsively displaced within 450 fs. It vibrationally cools inside the high spin state within 2.4 ps. **b** For AZA, the ligand structural rearrangement is statistically distributed during IVR, resulting in a broader distribution of structures, which narrows over 2.3 ps as IVR activates ligand motions and further molecular reorganization.

The reaction coordinates represent relative atomic motions from the initial LS state (*ξ*_1_, *ξ*_2_, *ξ*_3_, *ξ*_4_) = (2.0 Å, 3.3 Å, 82°, 82°) toward our simulated final HS state (2.2 Å, 3.5 Å, 85°, 85°). Overall, the Fe–N bonds have undergone a cumulative 0.2 Å elongation and 3° rotation. At the plateau associated with the photoinduced HS state (between +5 ps and +20 ps), the optimized values are: (2.19 ± 0.04 Å, 3.47 ± 0.04 Å, 84.9 ± 0.1°, 84.5 ± 0.1°) (see Supplementary Table 3). The first relaxation process is found to be comprised of an initial 0.15-Å and 2.2° change in the Fe–N bond lengths (*ξ*_1_) and angles (*ξ*_3_) respectively during the 450-fs ultrafast step. A further 0.05-Å elongation and 0.8° rotation of the same parameters can be observed during the subsequent IVR and vibrational cooling to the final fully relaxed HS structure. The ligand distances (*ξ*_2_) and angles (*ξ*_4_) to the central Fe(II) atom change by only 0.1 Å and 1.4° respectively during the first ultrafast step, with additional changes of 0.07 Å and 1.1° during the latter phases. These results are schematically shown in Fig. 5a.

The present observations of single-crystal BPY show that the local Fe–N bond motions are similar to those previously reported in solution^37^, while those of the ligands are different. We postulate that the latter is caused by the small unit cell of the LS state and the resulting chemical pressure from neighboring unit cells, which would inhibit reorganization of the photoexcited molecule^51^. Here, the ligands are observed to move simultaneously with the initial Fe–N bond elongations, and eventually expand to a volume that is ~20% smaller than that found in solution^37^. The net motions lead to lattice expansion which would experience a barrier to displacement from the lattice minimum determined by the surrounding unexcited molecules. In addition, the relative expansion of the ligand shell is smaller than the local Fe–N bond elongation in the initial ultrafast step; this gives a curved trajectory in the space defined by Fe–N motion and ligand motions (see Fig. 5a). Given that the ligands in BPY (2-2^’^-bipyridine) are much smaller, more rigid, and highly symmetrical in comparison with those of many other Fe(II) compounds (e.g., AZA), we postulate that the initial motions of ligands in SCO compounds with larger, more flexible ligands would be smaller, involve more nuclear degrees of freedom, and be spatially more distributed through IVR processes. The relaxation to the minimum in the HS state, in this case, is observed to be dominated by vibrational cooling processes to localize the net nuclear motions that map on to the HS potential surface.

As depicted in Fig. 5, we observe markedly different structural dynamics for BPY than in our previous UED study of another Fe(II)-based SCO compound, AZA^38^. In the latter case, the observed evolution of Bragg peak intensity followed a monoexponential decay behavior with a 2.3-ps time constant; this is fully described by structural dynamics wherein all motions—the Fe–N bond elongations, ligand butterfly motions, and local unit cell expansions—are coupled. The relevant process on this timescale is the relaxation of the vibrationally hot HS molecules via IVR and vibrational cooling through energy exchange with the surrounding lattice, leading to a narrowing of the Fe–N bond-length distribution. In contrast, a biexponential decay is observed in BPY. The slower 2.4-ps time constant is very similar to that observed in AZA and is assigned to a similar vibrational cooling process to arrive at the final fully relaxed HS structure.

It is important to note that the potential energy surfaces of the excited states and subsequent radiationless spin transition dynamics of BPY and AZA are similar but not identical^26,29,41^ due to their differing ligand-field strength. Following the arrival of the BPY molecule onto the HS state surface, the initial Fe–N bond distribution is narrower than that of AZA upon similar photoexcitation, and evolves in a more coherent manner, as observed by X-ray spectroscopies^26,30,40^. The number of low-frequency modes (of which there are many more in AZA than in BPY), the total number of degrees of freedom, and the symmetry of the ligands are important during the vibrational cooling process^13,19,52^. The delocalized low-frequency modes of BPY and AZA have been calculated using TD-DFT^53^ (details in Supplementary Note 7). The Fe–N bond elongation corresponds to a breathing mode of the Fe–N bond at ~150 cm^−1^^26^. Supplementary Fig. 4 shows the calculated normal modes in the frequency range from 0 to 400 cm^−1^ for three SCO molecules: BPY, AZA, and [Fe^II^(phen)_2_](NCS)_2_ (phen = 1,10-phenanthroline) (PHEN). PHEN is an SCO complex where the ligands are of a size in between those of BPY and AZA, and it has previously been compared to BPY^26^. While BPY and PHEN have a similar number of low-frequency modes (29 and 30, respectively), AZA has almost twice the number of low-frequency modes (49), reflecting its larger size and lower symmetry. It is this difference that gives rise to the broader distribution of displacements observed in AZA during nuclear reorganization.

Additionally, the ligands of BPY are centrosymmetric while those of AZA are of a lower symmetry; this results in strong coupling to only a few degenerate bath modes during the ISC crossing via FC active modes. Consequently, this difference leads to a more statistically averaged relaxation process in AZA that localizes the principal nuclear motions on the vibrational cooling time scale. As such, the structural changes associated with the ISC process of BPY are more strongly correlated, less spatially distributed, and could be observed by UED with the additional time constant of 450 fs. The difference in molecular reorganization processes between AZA and BPY can be further explained as the result of the significantly different HS potential energy surfaces. This difference manifests itself as distinct starting points for ISC and relative degrees of anharmonic coupling between the vibrational modes defining the nuclear reorganization pathways in the two cases. In this light, we have observed the coupling between the photoexcited electronic spin dynamics of the metal center and the coordinating ligands by direct observation of the nuclear reorganization using UED in conjunction with electronic information from X-ray spectroscopy work to assign the spin states involved (see Supplementary Discussion 1).

The complete structural dynamics associated with photoinduced SCO in single-crystal BPY have been directly characterized using UED. The changes in Bragg peak intensity display a global biexponential behavior that is attributed to coupled motions of the Fe–N bond and the ligands activated during SCO. Correlated bond elongation and dephasing of localized vibrational modes are assigned to the fast time constant. Then, a slower nuclear reorganization during vibrational cooling towards the potential minimum of the HS state, is reflected in additional motion in the atomic coordinates. By modeling four key structural dynamical groups (*ξ*_1_, *ξ*_2_, *ξ*_3_, *ξ*_4_), the complete structural dynamics of the photoinduced LS–HS transition are resolved. The ultrafast Fe–N bond elongation and further molecular reorganization suggested by previous indirect measurements are now observed with atomic resolution on the femtosecond timescale. These motions project out the molecular reorganization driven by transitions to different electronic surfaces and the spin correlated changes in the electron distribution. In this respect, it is noteworthy that the observed and relatively localized changes in nuclear configuration map onto the expected changes in electron density (Fig. 1b). Such changes in molecular structure towards the final fully relaxed HS state reflect the effects of spin on the electron correlation energies that lead to the occupation of the Fe(II) metal-centered half-filled d^6^ antibonding orbitals of BPY—a direct observation of Hund’s rule^9^ in action.

## Methods

### UED experiment

The excitation conditions used in our UED experiments are calibrated to be consistent with those applied in a previous study on single crystal [Fe^II^(bpy)_3_](PF_6_)_2_ using femtosecond optical spectroscopy^41^. Pump pulses centered at 400 nm are used with a low pump fluence of 5.12 mJ cm^−2^ (or 85 GW cm^−2^) to excite the sample within the single-photon regime^26,41^. This fluence is the linear range of the fluence dependent measurement. The excitation fraction is estimated to be 34% based on X-ray data^50^ and transient absorption measurements^15,41^. The repetition rate is set to 50 Hz to allow full recovery of the sample from the excited state back to the LS ground state and to avoid accumulated heat in the sample (see Supplementary Method for more details on experimental conditions). In this time resolved electron diffraction experiment, a 95-keV hybrid DC-RF electron diffraction setup is used to study the ultrafast atomic motion during the photoinduced phase transitions. The electron pulses were set to be 7 × 10^4^ electrons per pulse with a spot size of (100 ± 20)-μm diameter at the sample position, a repetition rate of 50 Hz, and 0.3% electron pulse shot to shot brightness stability. The instrument response function duration of the UED system has been measured to be (270 ± 30) fs FWHM by multiple methods^14,54^. Several techniques are applied to increase the signal-to-noise ratio; these include improving the quality of the diffraction patterns by varying sample thickness and crystal orientation. Ultra-microtomy was used to cleave the single-crystal BPY to ~150 nm, after optimizing the thickness in order to obtain the best excitation fraction and signal-to-noise ratio. The crystals are then picked from the water surface with a Cu 200-mesh TEM grid coated with amorphous carbon. The orientation of the sample has been indexed to be (2 1 0).

### Theory

TD-DFT as implemented in the Gaussian 09 package (Gaussian 09, 2009)^53^ was used for obtaining the natural transition orbitals (NTO) of the LS and HS state of [Fe^II^(PM-AzA)_2_(NCS)_2_], [Fe^II^(bpy)_3_](PF_6_)_2_, and [Fe^II^(phen)_2_(NCS)_2_] starting from geometry-optimized molecules with the B3LPY/LanL2DZ hybrid functional–basis set. Figure 1b shows the electron density difference during the SCO processes. Supplementary Fig. 2 shows the calculated low frequency modes of three SCO systems.

## Supplementary information

Supplementary Information

Description of Additional Supplementary Files

Supplementary Movie 1

## Data Availability

All raw images and source data are available from the authors upon reasonable request. The crystallographic coordinates for structures reported in this study have been deposited at the Cambridge Crystallographic Data Centre (CCDC), under deposition numbers 1972572 (excited high spin state structure calculated from X-ray data and structural model) and 1972373 (excited high spin state structure refined from ultrafast electron diffraction data). These data can be obtained free of charge from The Cambridge Crystallographic Data Centre via www.ccdc.cam.ac.uk/data_request/cif.

## Code availability

The source code is available from the authors upon reasonable request.

## References

[1] Hauser A. in *Spin Crossover in Transition Metal Compounds II. Topics in Current Chemistry*, Vol 234. (Springer, 2004).

[2] Chergui M, Collet E (2017). Photoinduced structural dynamics of molecular systems mapped by time-resolved X-ray methods. Chem. Rev..

[3] Halcrow MA (2007). The spin-states and spin-transitions of mononuclear iron(II) complexes of nitrogen-donor ligands. Polyhedron.

[4] Hauser A. & Reber C. in *50 Years of Structure and Bonding —The Anniversary Volume. Structure and Bonding* (ed. Mingos D.) Vol 172 (Springer, 2016).

[5] Yun S (2018). New-generation integrated devices based on dye-sensitized and perovskite solar cells. Energy Environ. Sci..

[6] Dirac PAM (1928). The quantum theory of the electron. Proc. R. Soc. A Math. Phys. Eng. Sci..

[7] Marian CM (2012). Spin-orbit coupling and intersystem crossing in molecules. Wiley Interdiscip. Rev. Comput. Mol. Sci..

[8] Herzberg, G. & Spinks, J. W. T. *Atomic Spectra and Atomic Structure*. (Dover Publications, 1945).

[9] Hund F (1927). Zur Deutung der Molekelspektren. II. Z. f.ür. Phys..

[10] Engel, T. & Reid, P. *Physical Chemistry*. (Pearson Benjamin Cummings, 2006).

[11] Sousa C (2013). Ultrafast deactivation mechanism of the excited singlet in the light-induced spin crossover of [Fe(2,2'-bipyridine)_3_]^2+^. Chem. - A Eur. J..

[12] Zhang Y, Bennett K, Mukamel S (2018). Monitoring ultrafast spin crossover intermediates in an Iron(II) complex by broad band stimulated X-ray Raman spectroscopy. J. Phys. Chem. A.

[13] Pápai M, Vankó G, Rozgonyi T, Penfold TJ (2016). High-efficiency iron photosensitizer explained with quantum wavepacket dynamics. J. Phys. Chem. Lett..

[14] Gao M (2013). Mapping molecular motions leading to charge delocalization with ultrabright electrons. Nature.

[15] Zhang W (2014). Tracking excited-state charge and spin dynamics in iron coordination complexes. Nature.

[16] McCusker JK (2019). Electronic structure in the transition metal block and its implications for light harvesting. Science.

[17] Chergui M (2015). Ultrafast photophysics of transition metal complexes. Acc. Chem. Res..

[18] Chergui M (2012). On the interplay between charge, spin and structural dynamics in transition metal complexes. Dalton Trans..

[19] Zhang W (2017). Manipulating charge transfer excited state relaxation and spin crossover in iron coordination complexes with ligand substitution. Chem. Sci..

[20] Kjær KS (2019). Luminescence and reactivity of a charge-transfer excited iron complex with nanosecond lifetime. Science.

[21] Marino A (2014). The role of ligand-field states in the ultrafast photophysical cycle of the prototypical iron(II) spin-crossover compound [Fe(ptz)_6_](BF_4_)_2_. Angew. Chem. - Int. Ed..

[22] Gawelda W (2007). Structural determination of a short-lived excited iron(II) complex by picosecond X-ray absorption spectroscopy. Phys. Rev. Lett..

[23] Lemke HT (2017). Coherent structural trapping through wave packet dispersion during photoinduced spin state switching. Nat. Commun..

[24] Lemke HT (2013). Femtosecond X-ray absorption spectroscopy at a Hard X-ray free electron laser: application to spin crossover dynamics. J. Phys. Chem. A.

[25] Bressler C (2009). Femtosecond XANES study of the light-induced spin crossover dynamics in an iron(II) complex. Science.

[26] Auböck. G, Chergui M (2015). Sub-50-fs photoinduced spin crossover in [Fe(bpy)_3_]^2+^. *Nat*. Chem.

[27] Consani C (2009). Vibrational coherences and relaxation in the high-spin state of aqueous [Fe^II^(bpy)_3_]^2+^. Angew. Chem. - Int. Ed..

[28] Cammarata M (2014). Sequential activation of molecular breathing and bending during spin-crossover photoswitching revealed by femtosecond optical and X-ray absorption spectroscopy. Phys. Rev. Lett..

[29] Marino A (2016). Activation of coherent lattice phonon following ultrafast molecular spin-state photo-switching: a molecule-to-lattice energy transfer. Struct. Dyn..

[30] Kjær KS (2019). Finding intersections between electronic excited ultrafast X-ray scattering and spectroscopy. Chem. Sci..

[31] Uemura H, Okamoto H (2010). Direct detection of the ultrafast response of charges and molecules in the photoinduced neutral-to-ionic transition of the organic tetrathiafulvalene-p-chloranil solid. Phys. Rev. Lett..

[32] Kawakami Y (2009). Optical modulation of effective on-site coulomb energy for the Mott transition in an organic dimer insulator. Phys. Rev. Lett..

[33] Först M (2011). Nonlinear phononics as an ultrafast route to lattice control. Nat. Phys..

[34] Khakhulin D (2019). Visualizing the coordination-spheres of photoexcited transition metal complexes with ultrafast hard X-rays. Phys. Chem. Chem. Phys..

[35] Britz A (2020). Resolving structures of transition metal complex reaction intermediates with femtosecond EXAFS. Phys. Chem. Chem. Phys..

[36] Collet E (2012). 100 Picosecond diffraction catches structural transients of laser-pulse triggered switching in a spin-crossover crystal. Chem. - A Eur. J..

[37] Lawson Daku LM, Hauser A (2010). Ab initio molecular dynamics study of an aqueous solution of [Fe(bpy)_3_](Cl)_2_ in the low-spin and in the high-spin states. J. Phys. Chem. Lett..

[38] Jiang Y (2017). Structural dynamics upon photoexcitation in a spin crossover crystal probed with femtosecond electron diffraction. Angew. Chem. - Int. Ed..

[39] Freyer B (2013). Ultrafast inter-ionic charge transfer of transition-metal complexes mapped by femtosecond X-ray powder diffraction. J. Chem. Phys..

[40] Marino Andrea, Servol Marina, Bertoni Roman, Lorenc Maciej, Mauriac Cindy, Létard Jean-François, Collet Eric (2013). Femtosecond optical pump–probe reflectivity studies of spin-state photo-switching in the spin-crossover molecular crystals [Fe(PM-AzA)2(NCS)2]. Polyhedron.

[41] Field R, Liu LC, Gawelda W, Lu C, Miller RJD (2016). Spectral signatures of ultrafast spin crossover in single crystal [Fe^II^(bpy)_3_](PF_6_)_2_. Chem. - A Eur. J..

[42] Bertoni R (2012). Femtosecond spin-state photoswitching of molecular nanocrystals evidenced by optical spectroscopy. Angew. Chem. - Int. Ed..

[43] Bertoni R (2016). Elastically driven cooperative response of a molecular material impacted by a laser pulse. Nat. Mater..

[44] Wolf MMN (2008). Sub-picosecond time resolved infrared spectroscopy of high-spin state formation in Fe(ii) spin crossover complexes. Phys. Chem. Chem. Phys..

[45] Van Kuiken BE (2016). Time-resolved X-ray spectroscopy in the water window: elucidating transient valence charge distributions in an aqueous Fe(II) complex. J. Phys. Chem. Lett..

[46] Baum P, Yang D, Zewail AH, Zewail H (2007). 4D Visualization of transitional structures in phase transformations by electron diffraction. Science.

[47] Liu LC (2017). Ultrafast electron diffraction study of single-crystal (EDO-TTF)_2_SbF_6_: Counterion effect and dimensionality reduction. Chem. Phys. Lett..

[48] Ishikawa T (2015). Direct observation of collective modes coupled to molecular orbital-driven charge transfer. Science.

[49] Zou, X., Hovmöller, S. & Oleynikov, P. *Electron Crystallography: Electron Microscopy and Electron Diffraction*. (Oxford University Press, 2011).

[50] Dick S (1998). Crystal structure of tris(2,2’-bipyridine)iron(II) bis(hexafluorophosphate), (C_10_H_8_N_2_)_3_Fe(PF_6_)_2_. Z. Kristallogr. - N. Cryst. Struct..

[51] Hauser A (2002). Chemical Pressure. Chim. Int. J. Chem..

[52] Rubtsov IV, Burin AL (2019). Ballistic and diffusive vibrational energy transport in molecules. J. Chem. Phys..

[53] Frisch, M. J. et al. *Gaussian 09* (Gaussian, Inc., Wallingford CT, 2009).

[54] Gao M, Jiang Y, Kassier GH, Miller RJD (2013). Single shot time stamping of ultrabright radio frequency compressed electron pulses. Appl. Phys. Lett..

