# Supplementary material for: Direct observation of nuclear reorganization driven by ultrafast spin transitions
Source: Nat Commun. 2020 Mar 23;11:1530. doi: 10.1038/s41467-020-15187-y (PMC7090058; doi:10.1038/s41467-020-15187-y)
Supplement: Supplementary file 1 — Supplementary Information [file 41467_2020_15187_MOESM1_ESM.pdf]

Supplementary Information

Direct Observation of Nuclear Reorganization Driven by Ultrafast Spin Transitions

Jiang *et al.*

Supplementary Methods – Experimental conditions

The single crystal of tris(bipyridine)iron(II) chloride $[Fe(bpy)_3](PF_6)_2$ (BPY) was prepared by Wojciech Gawelda.[1] Ultra-microtomy was used applied to cleave the single crystal BPY to be approximately 150 nm thick, after optimizing the thickness in order to obtain the best excitation fraction and signal-to-noise ratio. The crystals are then picked by from the water surface with a Cu 200-mesh TEM grid coated with amorphous carbon. The orientation of the sample has been indexed to be (2 1 0).

In this time resolved electron diffraction experiment, a 95-keV hybrid DC-RF electron diffraction setup is used to study the ultrafast atomic motion during the photoinduced phase transitions. The electron pulses were set to be 7×10^4 electrons per pulse with a spot size of (100 ± 20)-μm diameter at sample position, a repetition rate of 50 Hz, and 0.3% electron pulse shot to shot brightness stability. The time resolution of this femtosecond electron diffraction setup was characterized as (270 ± 30) fs FWHM by methods described in previous studies.[2–4]

The experiment conditions used in this experiment were set to be consistent with those applied in a previous femtosecond optical spectroscopy study of single crystal BPY.[1] At the sample position, 60-fs pump pulses centered at 400 nm were focused to be (575 ± 20) μm FWHM. The incident excitation fluence was 5.12 mJ cm^{-2} or 85 GW cm^{-2}. This fluence is the linear range of the fluence dependent measurement.[1] The excitation fraction was 34% based on the crystal information[5] and absorption measurements[1,6].

Supplementary Note 1 – Electron density difference during spin crossover

Supplementary Figure 1 is the electron density differences (EDD) between low spin (LS) and high spin (HS) states based on our calculations. The HS structure from the Gaussian[7] calculation was adopted for both LS and HS states reconstructions since the atomic position must be exactly the same for EDD. Then the electron density for LS and HS states was calculated by Gaussian[7]. The major difference between them is that LS is a singlet state and HS is a quintet state. In the end, the EDD between LS and HS was calculated by Multiwfn[8]. Supplementary Figure 1a is the 3D plot of EDD by VMD[9]. Blue is considered negative, whereas red is positive. Supplementary Figure 1b is the contour map of fragmental EDD in the plane of N-Fe-N by Multiwfn[8]. Solid line means positive and dash line means negative. During spin crossover (SCO), two paired electrons enter e_g orbitals as unpaired electrons[10]. Therefore, we should expect a decrease of *zx*, *yz*, *xy* orbitals density and increase of x^2-y^2 and z^2-r^2 orbitals density.

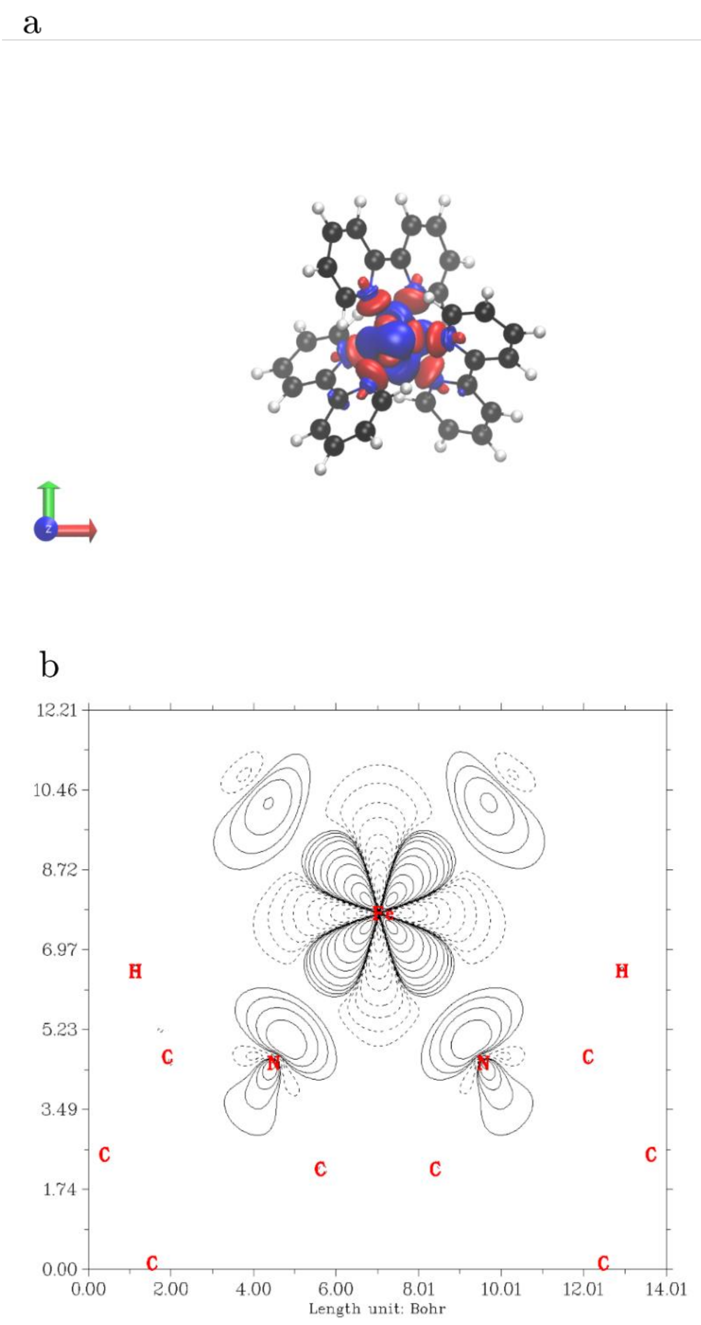

Supplementary Figure 1: Simulated electron density difference during SCO. (a) the 3D plot of electron density difference. Red denotes positive value of electron density differences during SCO, and blue denotes negative value of electron density differences (b) the contour map of fragmental EDD in the plane of N-Fe-N. Solid line means positive and dash line means negative.

Supplementary Note 2 – The relevance of direct observation of nuclear motions involved in spin transitions.

The nuclear motions are in response to spin transitions leading to a change in electron distribution. One could argue that spectroscopic probes are more sensitive to changes in electron distribution in terms of the relative effect on spectral changes associated with spin transitions. In fact, it is the change in spectra that allow assigning the observed dynamics to certain spin states, using various criteria to assign to specific spin states. However, theory is still needed to give insight into the associated changes in electron distribution. It is not a direct observable of changes in the electron distribution per se. Most important, the observed dynamics result from the very nuclear reorganization that is the direct observation of the femtosecond electron diffraction studies presented in this paper. The observed nuclear motions in fact provide unique probes of the changes in electron distribution and light up where these changes occur. Further, it is the ensuing nuclear relaxation processes that make the spin transition irreversible and lead to the lowest energy nuclear configuration defined by the potential energy of the high spin state. The nuclear reorganization convolved to the spin transition is typically depicted schematically as "wiggly arrows" to show the nonradiative relaxation to the lowest energy spin state. We can now see the specific details of these nuclear motions directly. In comparing the observed nuclear reorganization to the expected changes in electron distribution (Supplementary Figure 1), it is now apparent that the key motions for BPY are highly localized to the predicted changes in electron distribution that is in turn localized at the metal centered orbitals. This correlation is remarkably clear now and it does not involve just the Fe-N bond elongation but coupling of motions extending out to the ligands. This process is very similar to electron transfer in which nuclear reorganization stabilizes the associated change in electron distribution. The nuclear relaxation processes define the reaction coordinate. In

fact, spin transitions can likewise be treated within a reaction coordinate basis to depict the relaxation process along key modes. We can now see clearly the correlation between changes in electron density and processes leading to the enormous reduction in the number of nuclear degrees of freedom involved in directing structural. We expect this new insight to be transferrable to structural changes in general and provide a basis for predicting in advance key reaction modes directing chemistry.

Supplementary Note 3 – Calculation of HS state structure and simulation of electron diffraction patterns.

The initial low spin (LS) structure in single crystal has been measured by X-ray diffraction[5], while most theory calculation and experimental measurements of the final high spin (HS) state structure have been focused on the liquid environment[11,12]. In this work, the HS structure in solid state was calculated based on previous simulations of the HS structure in liquid. Our calculation was simplified by selecting four key motions, Fe–N bond elongation ξ_1, Fe–ligand elongation ξ_2, Fe–N rotation ξ_3, and Fe–Ligand rotation ξ_4 to present the structural dynamics during SCO, based on the good symmetry of single crystal BPY and previously reported studies of structure dynamics of SCO[13,14] (See Supplementary Figure 1a). The Fe–N bond elongation ξ_1 is the direct structural result of SCO dynamics due to weaker Fe–N bonding in the HS state. Since covalent bonds in ligands are much strong than coordinate bonds between Fe atom and six N atoms, the Fe–N rotation ξ_3 is believed to be strongly coupled to the initial Fe–N bond elongation. Ligand elongation ξ_2 and ligand rotation ξ_4 are introduced to offer additional freedom of movement to ligand, since molecule reorganization has been suggested by previous studies.[13,15] See Appendix I for our calculated HS structure by previous simulation and X-ray data.

Supplementary Table 1 summarizes the averages of selected geometric parameters. In the two spin states, the average values derived from our calculated structure of the HS state in single crystal are close to their values from previous simulations and available experimental data.

The experimental changes of Bragg peaks induced by photoexcitation are compared with simulated structure factor differences using the LS structure from static X-ray diffraction [5] and our simulated HS state structure based on previous simulations [12]. Structure factor calculations are used for these two structural end points with respect to spin state to show the intensity changes of Bragg peaks associated with atomic motions during SCO. The atomic positions within the unit cell determine the intensity of a given diffraction peak, which is proportional to the square of the structure factor.[16]. Then 3D structure factor differences between the LS structure from static X-ray diffraction and the simulated HS state based on prior simulations from Daku *et al*. [12] are calculated.. The 3D structure factor difference map is flattened into a 2D image using same crystal orientation obtained by indexing experimental electron diffraction patterns.

Figure 2d in the main article is the structure factor difference map between the LS and the HS in single crystal, simulated using the LS structure from static X-ray diffraction and our simulated HS state. The observed changes in Bragg peaks induced by photoexcitation (Figure 2b in the main article) are very close to the expected Bragg peak changes from our calculation. Therefore, the ultrafast changes in the Bragg peaks observed in our experiment can be attributed to the SCO dynamics.

		This work(S)		XRD(S)[c]	SIM1(L)[d]	SIM2(L)[e]	EXAFS(L)[f]
		CAL(S)[a]	FED(S)[b]				
Fe–N	LS			1.967	1.982	1.997	1.98

	HS	2.164	2.160		2.173	2.208	2.18
C2–C2'	LS			1.471	1.466	1.468	
	HS	1.425	1.465		1.481	1.486	
N–Fe–N'	LS			81.8	81.5	81.0	
	HS	75.846	75.806		75.7	75.0	
N–C2–C2'–N'	LS			6.4	0.6	3.0	
	HS	6.968	6.840		4.0	-0.4	

Supplementary Table 1: Averages of Selected Structural Parameters for $[Fe(bpy)_3]^{2+}$ in the LS and HS state. (a) Calculation of single crystal HS state from this work. (b) Measurement of photoinduced single crystal HS state from this work. (c) X-ray diffraction data of single crystal LS state. (d) Simulation of LS and HS in liquid. (e) Simulation of LS and HS in liquid. (f) EXAFS data of liquid HS of LS.

Supplementary Note 4 – Global fitting of time-dependent diffraction intensity changes of all bright diffraction peaks

During data analysis, the time-dependent diffraction intensity changes are best modelled by fitting a biexponential function with two time components, a fast component of 450 fs and a slow component with a time constant of 2.43 ps. On the other hand, in our previous ultrafast electron diffraction (UED) study on $Fe(PM\text{-}AzA)_2(NCS)_2$ (AZA), only one time constant was observed. Based on these results, we have a further discussion about the role of ligands in SCO. Therefore, it is important to perform very careful data analysis and support our observations.

All bright diffraction peaks in BPY and AZA UED studies are applied to fit simultaneously. The data points are fit using a global fitting model as follows:

$$S_i(t) = G_\sigma(t) \ast [c_i(1 - e^{-t/\tau})] = \frac{1}{2}c_i\left[\left(1 + \mathrm{erf}\left(\frac{t}{\sqrt{2}\sigma}\right)\right) - e^{-t/\tau}e^{\frac{1}{2}(\sigma/\tau)^2}\left(1 + \mathrm{erf}\left(\frac{t - \sigma^2/\tau}{\sqrt{2}\sigma}\right)\right)\right] \quad (1)$$

where σ is the instrument response time (267 fs FWHM or 113 fs 1/e). By fitting all the data points simultaneously, we obtained two time constants, 450-fs and 2.43 ps.

Supplementary Note 5 – Comparison between monoexponential and biexponential behaviour

In our BPY UED work, we observe two distinct time constants, while only one time constant was observed in our previous AZA UED work. In order to exemplify this difference, further data analysis was performed and reported herein.

Biexponential character of BPY

For biexponential character of BPY, it is often the case that biexponential fittings usually have better result than monoexponential fittings. To check whether a biexponential decay of τ_1 = 450 fs and $\tau_2 > \tau_1$ can be fit by a single monoexponential decay with $\tau_{1,2} \sim$ 2.32 ps, we simulated biexponential data $S_{\mathrm{sim}}(t, \tau_1, \tau_2)$ over a broad range of possible τ_1, τ_2 and applied our least-square fitting routine with $S_{\mathrm{fit}}(t, \tau_{1,2})$.

$$S_{\mathrm{sim}}(t, \tau_1, \tau_2) = G_\sigma(t) \ast \left[1 - \frac{1}{2}e^{-t/\tau_1} - \frac{1}{2}e^{-t/\tau_2}\right] \qquad \text{(S2)}$$

$$S_{\mathrm{fit}}(t, \tau_{1,2}) = G_\sigma(t) \ast [1 - e^{-t/\tau_{1,2}}] \qquad \text{(S3)}$$

In Supplementary Figure 2a and Supplementary Figure 2b, τ_1 is set to the instrument response time (450 fs), and τ_2 is adjustable from 0 ps to 6 ps. $\tau_{1,2}$ achievable is less than 1.2 ps, while one

time constant we observed in AZA is 2.3 ps. It is clear that no combination of τ_1 = 450 fs and τ_2 yields a signal that can be fit by a $\tau_{1,2}$ close to the observed value of 2.32 ps. Therefore, it is not possible that ultrafast time constant we observed in BPY UED work is simply obscured by fitting.

For BPY data analysis, time dependent intensity changes of Bragg peaks are fit by both monoexponential and biexponential functions in Supplementary Figure 2c. It is obvious that biexponential function can fit our signal better than monoexponential function. In Supplementary Figure 2d, root mean square error (RMSE) is showed for both monoexponential and biexponential fitting. Biexponential fitting has a smaller RMSE than any monoexponential fitting, regardless of the fit parameters. On the other hand, fitting three or more time constants may have better results than a biexponential fit, but the results cannot agree with the observed structural dynamics and time constant suggested by previous literature. With this, we can confidently say that only one time constant was observed for AZA, whereas two were clearly observed for BPY.

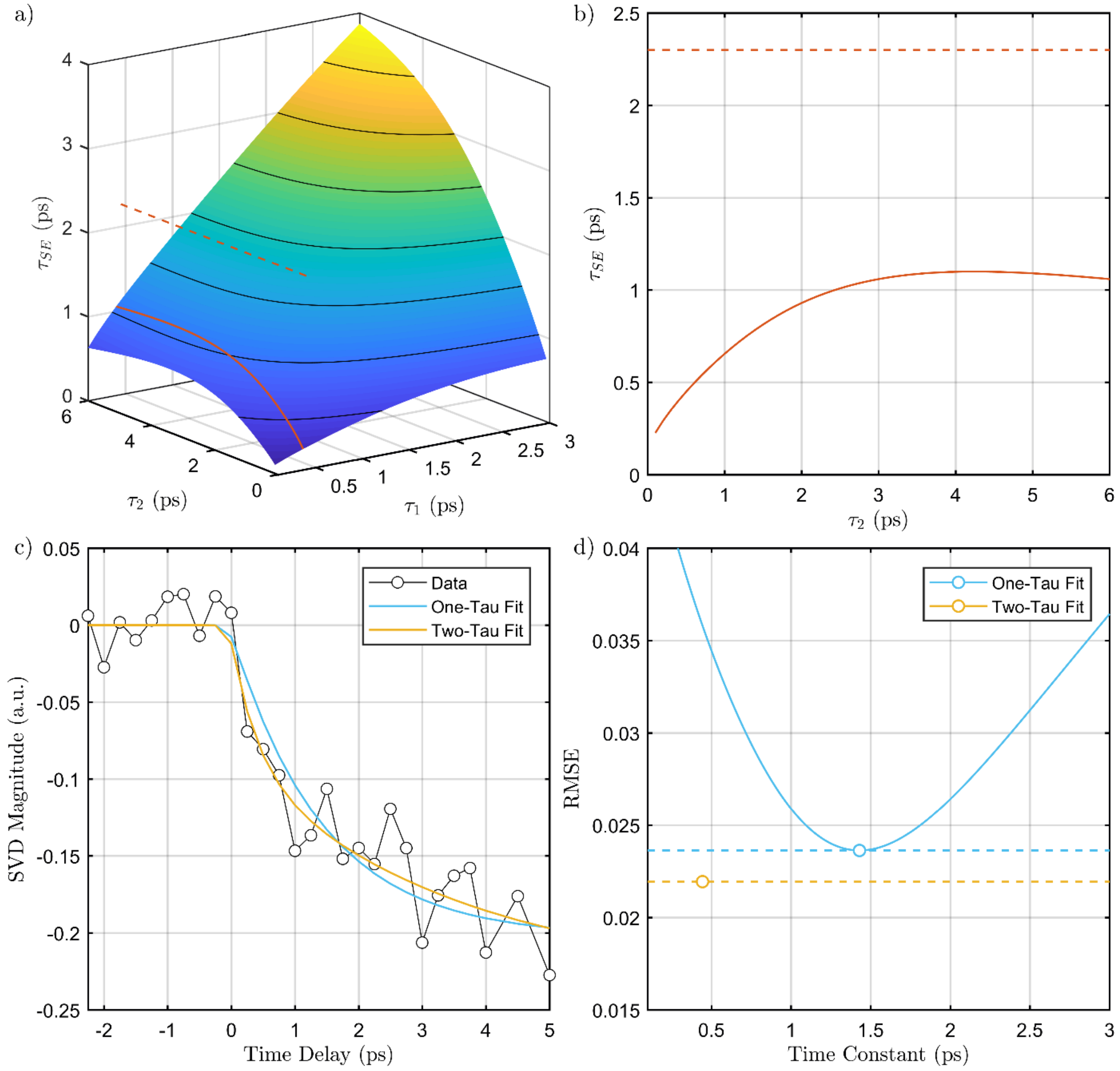

Supplementary Figure 2: Comparison between monoexponential and biexponential behaviour. (a) τ_1 is set to be 450 fs (instrument response time), τ_2 is adjusted from 0 ps to 6 ps. The red line represents a plot of fit monoexponential time constant as a function of τ_2 with τ_1 = 450 fs. The red dash line represents a 2.3-ps time constant obtained by monoexponential fitting. (b) 2D surface plot of monoexponential fitting over the possible values of τ_1 and $\tau_{2.}$ (c)

biexponential and monoexponential fittings of BPY signal. (d) root mean square error of both bi-exponential and mono-exponential fittings.

Monoexponential character of AZA

For monoexponential character of AZA, on the other hand, there is a concern that a fast signal could be present in our data and is simply obscured by fitting a possible biexponential decay with a single monoexponential function. The literature suggests that the Fe–N bond undergoes abrupt elongation on the same timescale as the spin transition, $\tau \sim 160$ fs[17]. However, the UED measurements on Fe(AZA) only show slower dynamics, where every time trace, from diffraction spots of any order, can be well fit by a single monoexponential function with time constant of ~ 2.3 ps[15]. With respect to the question about the time resolution, observable dynamics that are faster than the IRF time should still appear in the measured time traces, albeit as an additional monoexponential term with time constant $\tau_1 = \tau_{\mathrm{IRF}} * \tau_{\mathrm{fast}}$ on the top of the original fit with a slower time constant, $\tau_2 > \tau_1$. We need to keep in mind that we are not talking about changes in optical spectra. There must be significant structural changes associated with the putative missed fast relaxation dynamics for AZA. If there were structural changes within our signal to noise, i.e. comparable or even a factor of 10 smaller than the picosecond relaxation components, we would have to see the changes in IRF limited changes in structure factors, which we do not observe.

Notwithstanding, we examined this point more carefully. It can be shown that no monoexponential model with time constant τ_{m}= 2.3 ps can robustly fit to data that is really biexponential with such time constants τ_2 and τ_1 without ruining the monoexponential fit. Some Bragg peaks, such as $(1\bar{1}0)$, $(3\bar{3}0)$, and (002) for AZA, have contributions from the Fe–N mode and the ligand mode.

In Supplementary Figure 3, in the case of Brag peak $(3\bar{3}0)$, to check whether a bi-exponential decay of $\tau_1 = \tau_{\mathrm{IRF}}$ and $\tau_2 > \tau_1$ can be fit by a single mono-exponential decay with $\tau_{1,2} = 2.32$ ps, we simulated bi-exponential data $S_{\mathrm{sim}}(t, \tau_1, \tau_2)$ (Supplementary Equation 2)over a broad range of possible τ_1, τ_2 and applied our least-square fitting routine with $S_{\mathrm{fit}}(t, \tau_{1,2})$ (Supplementary Equation 3).

In Supplementary Figure 3a, τ_1 is set to be the instrument response time. The largest $\tau_{1,2}$ achievable is less than 0.8 ps. In Supplementary Figure 3b, the white line gives the combination of τ_1 and τ_2 that yields a signal that can be fit to 2.32 ps. Clearly, there is no combination of $\tau_1 <$ 1 ps (never mind the 270-fs IRF) and τ_2 that can yield a signal which can be fit by a $\tau_{1,2}$ close to the observed value of 2.32 ps. Given that we can resolve the specific amplitude of the nuclear motions involved in the 2.32-ps exponential relaxation process, we can state unequivocally that any fast structural changes are an order of magnitude smaller than the picosecond dynamics associated with vibrational cooling to the HS potential minimum that dominates the nuclear reorganization for this system. This statement is different than in the case of spectroscopic observables in which there are unknown differences in oscillator strengths for spectral changes associated with different electronic states. In UED, electron diffraction provides a state independent determination of atomic positions. There are obviously different electronic states and spin involved in the AZA spin/structural dynamics, but the nuclear reorganization is dominated by those motions driven in the HS manifold with rapid IVR uniformly distributing the ensuing dissipation processes till the final relaxation phase via vibrational cooling.

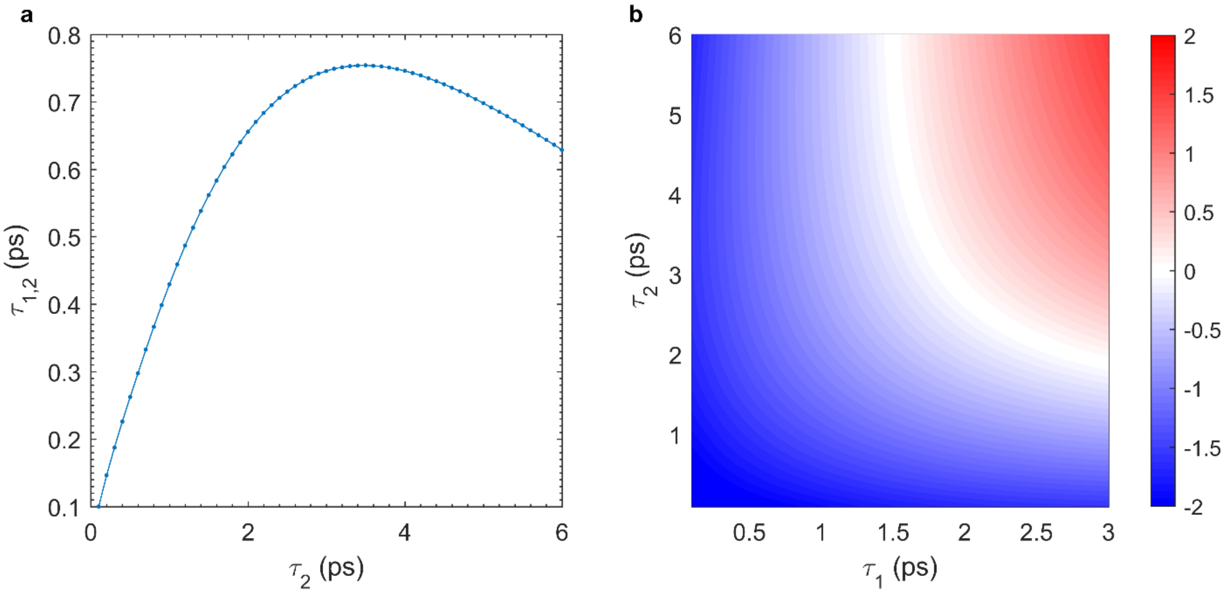

Supplementary Figure 3: Fitting a bi-exponential decay with a single mono-exponential function. (a) plot of fit mono-exponential time constant as a function of the τ_2 for τ_1 = 0.113 ps. (b) 2D surface plot of fit $\tau_{1,2} - 2.32$ ps over the possible values of τ_1 and τ_2.

Supplementary Note 6 – Time-dependent model of atomic motions

In this work, four key structural dynamics groups are selected to present the full structural dynamics of SCO Fe–N bond elongation ξ_1, Fe–ligand elongation ξ_2, Fe–N rotation ξ_3, and Fe–Ligand rotation ξ_4, based on the previous structural studies of SCO[13,15]. For each time point measured in the UED experiment, a least square fitting routine is used to find the optimal set of parameters of reaction coordinates that would best match the observed changes in diffraction intensity[2,15].

Supplementary Note 7 – Low-frequency modes of three related spin crossover systems

In this work, vibrational cooling processes and delocalized low frequency modes were discussed to investigate the role of the ligand in SCO. As shown in Supplementary Figure 4, TD-DFT as implemented through in the Gaussian 09 package was used to obtain low frequency modes of three related SCO samples, AZA, BPY, and $Fe(phen)_2(NCS)_2$ (PHEN). PHEN is used to a reference to compare BPY and AZA, since its ligand size between that of BPY and AZA and it has been compared previously to BPY when discussing the low frequency modes.

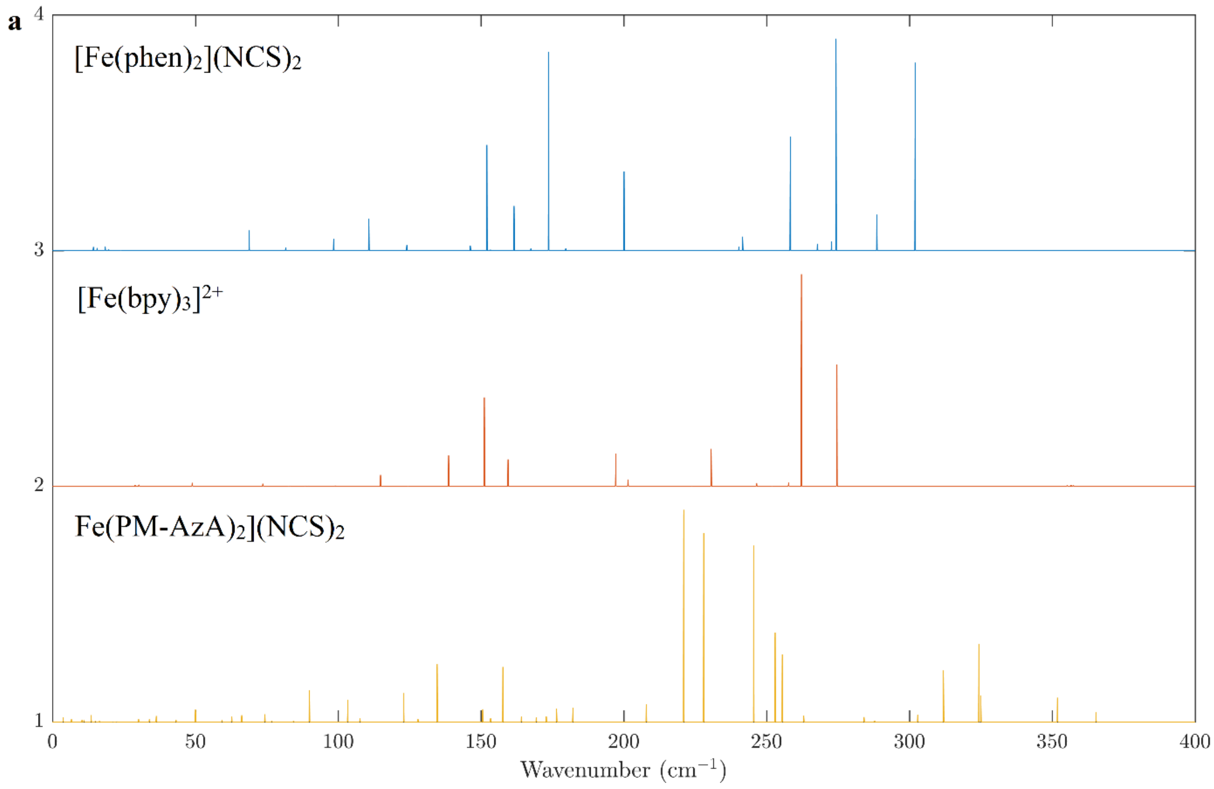

b

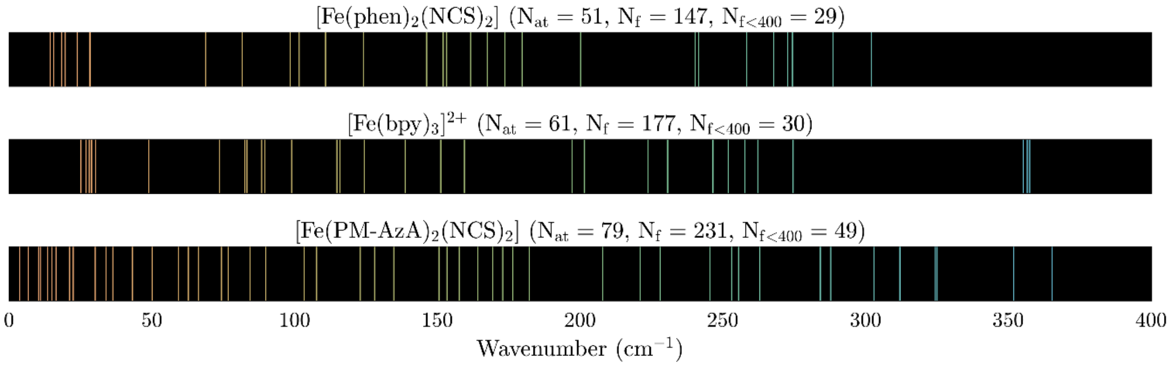

Supplementary Figure 4: Gaussian calculation of low frequency modes of three SCO system, PHEN, BPY, and AZA. (a) simulated IR spectrum and (b) spectral lines of the three SCO system.

Supplementary Discussion 1 – Discussion of experimental differences between ultrafast X-ray spectroscopy and electron diffraction

X-ray spectroscopies in solution, including X-ray absorption near edge structure (XANES), X-ray emission spectroscopy (XES), and X-ray diffuse scattering (XDS), and our present ultrafast electron diffraction (UED) are complementary tools to study structural dynamics. XES is more sensitive to electronic dynamics. XANES is rich in electronic structure information; however, the structural sensitivity of XANES is limited to Fe–N bond distance. XDS has limited structural sensitivity since the scattered signal is shaped by the dephasing of waves scattered by the different atoms of disordered molecules in solution. In contrast, UED is a direct measurement of atomic motions over the full extent of the molecule, which makes it possible to observe correlated motions driven over length scales defined by the change in potential energy surface. There are differences in sensitivity to particular motions with X-ray methods being most sensitive to motions local to

the metal center. These different probes are complementary and help provide consistency checks on interpretation. We note that our UED findings with respect to dynamics are in reasonable agreement with previous XES and XDS studies[18], however, there are slightly different time constants. These differences can be attributed to different sensitivities to specific motions, excitation conditions, and the previous discussed differences in solution phase and solid state on intermolecular coupling to the effective reaction coordinate[1]. In fact, the overall structure information from UED that encompasses the whole molecule, including ligand motions, is exactly what X-ray spectroscopies are also seeking. Towards this objective, extended X-ray absorption fine structure (EXAFS) has recently been applied to the study of the structural dynamics of a transition metal complex, although it is still limited by the signal-to-noise ratio and the tunable high-energy X-ray source[19].

We would like to emphasize that prior knowledge of the excited HS state structure is not necessary for our data analysis method. The crystallography used in our electron diffraction work has a higher spatial resolution compared to XDS studies of disordered molecules in solution. Due to a lack of structural sensitivity, Kjaer et al. linearly interpolates the Fe–N bond distances to model the structure dynamics probed by XDS, based on the knowledge of the initial LS and final HS structure[18]. Afterwards, the simulated scatter signals are compared to experimental signals. This modelling analysis can obtain the global best-fit structure to explain the experimental data but is limited by the assumption that the Fe–N bond coordinates moves linearly from ground state to simulated excited state without any torsions of ligands. This study also requires accurate structures of both the ground LS state and excited HS state, which are not necessarily known beforehand for many experiments.

In this respect, our approach to interpreting the results in uniquely different than Kjaer et al[18]. The time-dependent map of the molecular motions is achieved without the above simulated excited HS state structures. Our simulated single crystal HS state structure is only used to conclude that the observed ultrafast Bragg peak changes are due to photoinduced SCO (to confirm that the changes in Bragg peak intensities observed are consistent with the structure of the HS state). In our parameterized molecular model, four structural dynamics groups are selected based on basic chemical knowledge about the structure and the observed phase transition to avoid chemically unreasonable structures and reduce the total degrees of freedom – use of chemical intuition if you will for the system at hand. For example, the ligand is considered as one dynamic group to move together because of ligand rigidity[13,20]. These four parameterized molecular modes are computed independently to calculate the structure factor and compare it to the experimental data with the Pearson coefficient equation. This is because the structure factor squared is our experimentally observed parameter, directly related to the brightness of the Bragg peaks. Our data analysis technique can calculate different time constants pertaining to different structural modes. Thus, prior knowledge of the excited state structure is not necessary for this method. This is an important feature as the displacement of atomic positions during SCO may not move linearly between the ground state and the excited state structures. This refinement approach within the defined basis is not constrained by any assumptions for the structural evolution.

With our data analysis method, we have successfully resolved the structural dynamics of the photoinduced HS state of BPY in the single crystal state. Since there were no prior assumptions made, this approach allows us to observe differences in the BPY structural dynamics between solution and solid state. This unique new insight really emphasizes the differences between solution phase and solid state on the bath coupling, which we discuss in terms of differences in

chemical pressure, as presented in Figure 4 of the main manuscript. The remarkable achievement of this work is the resolution of the curved trajectory in both Fe–N motion and ligand motion space, as presented in Figure 5 of the main manuscript, which would not have been possible if we constrained the dynamics along a particular coordinate such as has been typically done.

Furthermore, the other important difference between our work and previous studies is the excitation fluence. Our work is in the single-photon excitation regime with no more than 5.12 mJ cm^{-2} (or 85 GW cm^{-2}) with a 34% excitation fraction of molecules in a ~150-nm thick crystal. Damage to the molecules in the single crystal was readily apparent above this fluence. In contract, the excitation fluence used by XES and XDS was reported to be 85 mJ cm^{-2} with an excitation fraction of 42% (averaged over the entire excitation volume). From the given values in these studies, the peak power at the sample was 1.9 TW cm^{-2} with an excitation fraction at the sample surface of 177%. This excitation fluence necessarily involves multiphoton absorption and the peak power is sufficiently high to lead to dominant ionization effects. Please see the recent paper[21]. At this peak power, with similar transition strengths multiphoton effects dominate and there are multiple excited state potentials and accessible reaction coordinates that will make separating the relevant motions involved in SCO difficult. We draw comparisons to this work but wish to note there are some open issues in extending this comparison any further than qualitative features.

Fractional coordinates of structure of $[Fe(bpy)_3](PF_6)_2$ calculated from X-ray data and structure model

Label	Element	x	y	z
Fe	Fe	0	0	0.25
N	N	0.2049	0.0851	0.1837
C1	C	0.2454	0.1677	0.1158
C2	C	0.3728	0.2092	0.0750
C3	C	0.4635	0.1583	0.1022
C4	C	0.4274	0.0775	0.1733
C5	C	0.2983	0.0420	0.2131
P	P	0.3333	0.6667	0.1302
F1	F	0.2258	0.6831	0.1792
F2	F	0.5660	0.2143	0.4211
H1	H	0.1811	0.2000	0.0948
H2	H	0.3991	0.2720	0.0278
H3	H	0.5501	0.1790	0.0720
H4	H	0.4922	0.0464	0.1952

Supplementary Table 2 – Fractional coordinates of structure of $[Fe(bpy)_3](PF_6)_2$ calculated from X-ray data and structure model. The unit cell parameters are from previous X-ray data[5]. The crystal information file (CIF) is available for download.

Fractional Coordinates of HS structure of $[Fe(bpy)_3](PF_6)_2$ refined from UED data

Label	Element	x	y	z
Fe	Fe	0.00	0.00	0.25
N	N	0.20391974	0.08590322	0.183151676
C1	C	0.242717717	0.168260785	0.115384698
C2	C	0.369777543	0.210407402	0.074060785
C3	C	0.461300137	0.160158082	0.100744984
C4	C	0.426421195	0.079414574	0.171834477
C5	C	0.297615816	0.043310125	0.212147757
P	P	0.3333	0.6667	0.1302
F1	F	0.2258	0.6831	0.1792
F2	F	0.566	0.2143	0.4211
H1	H	0.177765415	0.200052264	0.094723693
H2	H	0.395245663	0.273137804	0.02688149
H3	H	0.547718869	0.181371973	0.070146174
H4	H	0.491857718	0.048826275	0.193293008

Supplementary Table 3 – Fractional Coordinates of HS structure of $[Fe(bpy)_3](PF_6)_2$ refined from UED data. The unit cell parameters are adopted from previous X-ray data[5].The unit cell parameters are from previous X-ray data[5]. The crystal information file is avaible for download.

Supplementary Acknowledgment

We thank Beta Yu, Elizabeth Pinto, and Peach Tailor for their constant borkful support in the preparation of this manuscript.

Supplementary Reference

1. Field, R., Liu, L. C., Gawelda, W., Lu, C. & Miller, R. J. D. Spectral Signatures of Ultrafast Spin Crossover in Single Crystal $[Fe^{II}(bpy)_3](PF_6)_2$. *Chem. - A Eur. J.* **22**, 5118–5122 (2016).
2. Gao, M. *et al.* Mapping molecular motions leading to charge delocalization with ultrabright electrons. *Nature* **496**, 343–346 (2013).
3. Gao, M., Jiang, Y., Kassier, G. H. & Dwayne Miller, R. J. Single shot time stamping of ultrabright radio frequency compressed electron pulses. *Appl. Phys. Lett.* **103**, (2013).
4. Gao, M. *et al.* Full characterization of RF compressed femtosecond electron pulses using ponderomotive scattering. *Opt. Express* **20**, 12048 (2012).
5. Dick, S. Crystal structure of tris(2,2'-bipyridine)iron(II) bis(hexafluorophosphate), $(C_{10}H_8N_2)_3Fe(PF_6)_2$. *Zeitschrift für Krist.* - New Cryst. Struct. **213**, 356-356 (1998).
6. Zhang, W. *et al.* Tracking excited-state charge and spin dynamics in iron coordination complexes. *Nature* **509**, 345–348 (2014).
7. Frisch, M. J., et al. *Gaussian 09*, Gaussian, Inc., Wallingford CT (2009).
8. Lu, T. & Chen, F. Multiwfn: A multifunctional wavefunction analyzer. *J. Comput. Chem.* **33**, 580–592 (2012).
9. Humphrey, W., Dalke, A. & Schulten, K. VMD: Visual molecular dynamics. *J. Mol. Graph.* **14**, 33–38 (1996).
10. Hauser A. Light-Induced Spin Crossover and the High-Spin→Low-Spin Relaxation. In: *Spin Crossover in Transition Metal Compounds II. Topics in Current Chemistry,* vol **234**. (Springer, 2004).
11. Gawelda, W. *et al.* Structural analysis of ultrafast extended x-ray absorption fine structure with subpicometer spatial resolution: Application to spin crossover complexes. *J. Chem. Phys.* **130**, 124520 (2009).
12. Lawson Daku, L. M. & Hauser, A. Ab Initio Molecular Dynamics Study of an Aqueous Solution of $[Fe(bpy)_3](Cl)_2$ in the Low-Spin and in the High-Spin States. *J. Phys. Chem. Lett.* **1**, 1830–1835 (2010).
13. Cammarata, M. *et al.* Sequential Activation of Molecular Breathing and Bending during Spin-Crossover Photoswitching Revealed by Femtosecond Optical and X-Ray Absorption Spectroscopy. *Phys. Rev. Lett.* **113**, 227402 (2014).
14. Hauser, A., Enachescu, C., Daku, M. L., Vargas, A. & Amstutz, N. Low-temperature lifetimes of metastable high-spin states in spin-crossover and in low-spin iron(II) compounds: The rule and exceptions to the rule. *Coord. Chem. Rev.* **250**, 1642–1652 (2006).
15. Jiang, Y. *et al.* Structural Dynamics upon Photoexcitation in a Spin Crossover Crystal Probed with Femtosecond Electron Diffraction. *Angew. Chemie - Int. Ed.* **56**, 7130–7134 (2017).
16. Zou, X., Hovmöller, S. & Oleynikov, P. *Electron crystallography : electron microscopy and electron diffraction*. (Oxford University Press, 2011).
17. Lemke, H. T. *et al.* Coherent structural trapping through wave packet dispersion during

photoinduced spin state switching. *Nat. Commun.* **8**, 15342 (2017).
18. Kjær, K. S. *et al.* Finding intersections between electronic excited ultrafast X-ray scattering and spectroscopy. *Chem. Sci.* **10**, 5749-5760 (2019).
19. Britz, A. *et al.* Resolving structures of transition metal complex reaction intermediates with femtosecond EXAFS. *Phys. Chem. Chem. Phys.* (2019). DOI:10.1039/C9CP03483H
20. Collet, E. *et al.* 100 Picosecond Diffraction Catches Structural Transients of Laser-Pulse Triggered Switching in a Spin-Crossover Crystal. *Chem. - A Eur. J.* **18**, 2051–2055 (2012).
21. Nass Kovacs, G. *et al.* Three-dimensional view of ultrafast dynamics in photoexcited bacteriorhodopsin. *Nat. Commun.* **10**, 3177 (2019).
