# Supplementary material for: Direct observation of nuclear reorganization driven by ultrafast spin transitions
Source: Nat Commun. 2020 Mar 23;11:1530. doi: 10.1038/s41467-020-15187-y (PMC7090058; doi:10.1038/s41467-020-15187-y)
Supplement: Supplementary file 2 — Description of Additional Supplementary Files [file 41467_2020_15187_MOESM2_ESM.pdf]

Description of Additional Supplementary Files

File Name: Supplementary Movie 1
Description: Structural dynamics of photoinduced spin crossover in $[Fe^{II}(bpy)_3](PF_6)_2$ by ultrafast electron diffraction.
